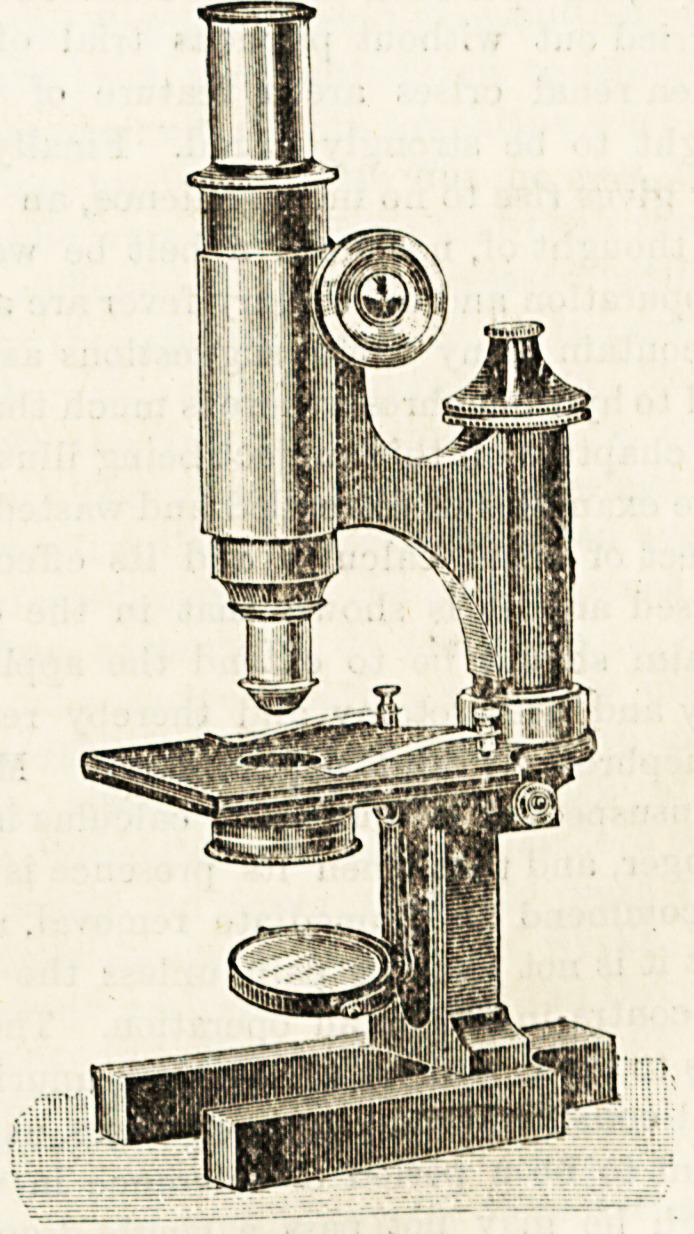# New Appliances and Things Medical

**Published:** 1901-11-09

**Authors:** 


### NEW APPLIANCES AND THINGS MEDICAL.

### ROSS' NEW STUDENTS' "STANDARD" MICROSCOPE.

(ROSS, LTD., 111 NEW BOND STREET, LONDON, W.)

THIS is a very good working microscope. It has a square blackened stage with the ordinary clips, a diagonal rack and pinion movement, and a direct-acting fine adjustment which is all that can be wished for in regard to accuracy and smoothness of working. There are both plane and concave mirrors, an Abbé condenser on a swing-out substage, and an iris diaphragm. The stand has an inclining limb and is very firm and steady. One eye-piece and two object glasses are supplied, the latter being of $\frac{2}{3}$ inch and of $\frac{1}{6}$ inch respectively. There is also an admirable triple-nose piece—one which seems to be really dust-tight. This is a great advantage. As we need hardly say in regard to anything coming from this firm, the optical part of this microscope is very good. Moreover, all the moving parts work very well and it will in every way be found a serviceable and useful students' microscope. The price is £10.

## Figures and Tables

**Figure f1:**